# Components of engagement in saying-is-believing exercises

**DOI:** 10.1007/s12144-022-02782-z

**Published:** 2022-02-03

**Authors:** Hye Rin Lee, Lisabeth M. Santana, Peter McPartlan, Jacquelynne S. Eccles

**Affiliations:** 1 School of Education, University of California, 3200 Education Bldg. , Irvine, CA 92697 USA; 2 Department of Physics and Astronomy, University of Pittsburgh, Pittsburgh, PA USA; 3 Department of Psychology, San Diego State University, San Diego, CA USA; 4 Institute for Positive Psychology & Education, Australian Catholic University, PO Box 968, North Sydney, NSW 2059 Australia

**Keywords:** Attitude change, Saying-is-believing, Video creation, Wise interventions

## Abstract

The saying-is-believing effect is an important step for changing students’ attitudes and beliefs in a wise intervention. However, most studies have not closely examined the process of the saying-is-believing effect when individuals are engaged in the activity. Using a qualitative approach, the present study uses an engagement framework to investigate (a) components of engagement in the saying-is-believing effect; and (b) how differently students may engage in a saying-is-believing exercise. Semi-structured interviews were conducted with 14 undergraduates in a scholarship program for low-income transfer students from community college. Analysis using inductive and deductive approaches found that students varied on the extent to which they experienced the effectiveness of the saying-is-believing effect through affective, cognitive, and behavioral experiences. The study offers examples of how people can indeed differ in the extent to which they experience the saying-is-believing effect, and the implications for designing more effective interventions. Specifically, students’ positive affective experiences from seeing the larger goal of creating videos may be important components for the saying-is-believing effect to work. Behavioral experiences, such as learning soft skills, academic skills learned indirectly from the intervention, and academic skills learned directly from the intervention were accompanied by both positive affective and cognitive experiences. Findings show the importance of students’ differential engagement in saying-is-believing exercises both for building more effective wise interventions and interpreting heterogeneity in intervention effectiveness.

## Introduction

A continual mission in the field of education is to keep students motivated and promote positive attitudes in school. In order to do so, psychologists have leveraged research on using attitudes and beliefs to influence behavior to support students’ achievement of their own educational goals (Ajzen & Fishbein, 2005; Fazio, 1986; Gormezano et al., 1987; Hecht et al., 2021). One rapidly growing relevant area of research within the intersections of social psychology and education is the work on wise interventions. Wise interventions in education aim to alter how people think or feel about themselves or the social situation in a brief, precise, and therefore low-cost way, to increase academic persistence (Walton, 2014; Walton & Wilson, 2018). For example, social belonging interventions focus on reducing threats of belonging for socially stigmatized groups in college (i.e., racial and ethnic minorities, women, first-generation students, etc.) by changing the way they think about their fit in college (Walton & Cohen, 2007, 2011). Instead of having students think that “people like me do not belong here,” they are asked to internalize the message, “everyone struggles in college and feelings of adversity get better over time.” This potentially simple change in students’ psychological interpretation of events can alter behavioral responses and create recursive processes that culminate in substantial improvements to academic outcomes (Cohen et al., 2009; Yeager & Walton, 2011).

By targeting psychological processes, wise interventions are often relatively low-cost, which can introduce both benefits and challenges. These interventions can be especially beneficial because they do not take much time for students, do not require extensive resources for the instructor, and therefore can be introduced into a wide variety of course or program curricula with relative ease. On the other hand, there is a very real challenge that, amidst a deluge of course content and other activities, students who receive these short interventions may not engage with them sufficiently enough to change their psychological processes. Students who listen to, but do not internalize the message, would then not be expected to translate the experience into psychological and behavioral changes.

One mechanism through which people are asked to engage with the message in such short interventions (e.g., mindset, difference education, personal values, etc.) is self-advocating for the message. In other words, individuals are asked to not only hear or read about these ideas, but also reaffirm the message of the intervention using their own words and advocate for it using their own personal experiences. Individuals may be first told, for example, that they are not the only ones struggling in college and this feeling is only temporary. However, passively consuming information has been bemoaned as a relatively weak form of changing student cognition (Emig, 1977; Mayer, 2003), and students may be inundated with academic information throughout a given day. Because of this, wise interventions deliberately design interventions to include methods that will help students engage with and better internalize their short motivational messages. A popular way of accomplishing this is subsequently asking students to relay this information to incoming first-year students through writing a letter or filming a video. When this process helps students engage with and internalize the message, the phenomenon is known as the saying-is-believing effect (Higgins & Rholes, 1978).

Educational wise interventions continue to be influential in changing students’ attitudes, which in turn help recruit and retain diverse individuals in various fields. In particular, they have been found to change various important outcomes, such as increased grade point average (Yeager et al., 2014), greater career satisfaction and psychological well-being (Brady et al., 2020), as well as more engagement in academic-related activities (Walton & Cohen, 2007). However, less is known about for whom the intervention is most effective, a topic that has received much attention as debates over wise interventions have sought to move from replicability (“does it work?”) to generalizability (“for whom does it work and why?”) (McPartlan et al, 2020; Tipton, 2019; Walton & Yeager, 2020; Yeager et al., 2019; Yeager & Dweck, 2020). This has required explaining heterogeneity in treatment effects.

In experimental studies, an important part of understanding heterogeneity in the effectiveness of wise interventions is the manipulation check, which seeks to determine whether students actually internalized the intended psychological belief. When conducted among large samples, this may be done immediately after the message is delivered by asking survey questions that measure whether the participant recalled the most central message of the intervention (e.g., Yeager et al., 2016). If the message cannot be remembered immediately after being exposed to it, the message itself is considered unlikely to have been internalized. But perhaps, more insight into whether the message was internalized can be investigated by measuring the targeted psychological processes immediately after the intervention. For instance, Yeager and colleagues (Yeager et al., 2016) asked participants to report on their anticipated feelings of belonging immediately after the social belonging intervention was delivered controlling for baseline and pre-intervention levels of belonging uncertainty. However, in studies that include saying-is-believing exercises, students’ own words might offer richer data through which to determine the extent to which students have internalized the message.

As researchers investigate mindset interventions in different populations and contexts, maintaining the integrity of the treatment itself is essential, and the saying-is-believing component is thought to be a crucial component of that integrity. Researchers have begun to use text-analysis programs such as the Linguistic Inquiry and Word Count (LIWC) program (Pennebaker et al., 2015) to analyze utility value essays. Some have confirmed that, as intended, students in the intervention conditions are engaging more with the content by writing more (Harackiewicz et al., 2016), writing about the content with more personal focus (“I” and “you” pronouns), and referencing social processes (Harackiewicz et al., 2016; Hecht et al., 2019; Priniski et al., 2019). Other than these, there are few studies that thoroughly analyze the extent to which students are engaging with the intended message through saying-is-believing exercises. As we look to understand heterogeneity in the effects of interventions, identify those who are not substantially benefitting, and improve intervention effectiveness, understanding heterogeneity in the effectiveness of saying-is-believing exercises can offer vital insights.

### Components That Engage Students in Saying-is-Believing Exercises

The saying-is-believing effect is widely cited as one of the primary reasons wise interventions work in the social psychological literature (Aronson et al., 2002; Stephens, et al., 2014; Walton & Cohen, 2007, 2011). This line of reasoning was primarily influenced by Higgins and Rholes’ (1978) study that found that individuals are more likely to internalize a message if they are put into a position of advocating for that very message. Common procedures that intervention creators use to induce the saying-is-believing effect are asking participants to write an essay, letter, or film a video about their personal experiences (Aronson et al., 2002; LaCosse et al., 2020; Walton & Cohen, 2011; Williams et al., 2020; Yeager et al., 2016). For instance, Walton and Cohen (2007, 2011) asked participants to not only read testimonials from upperclassmen about the normalcy of struggling in college during their first years and fitting in over time, but also to film a video for future college students about why experiences in college change over time, combining their own experiences with content provided from the intervention materials. Likewise, Stephens and colleagues (Stephens et al., 2014) had participants create a short video testimonial to incoming college students about what they learned in the intervention panel.

Within these exercises, students are often given specific, important requirements that outline how the task should be approached. Typical requirements include that the students should themselves advocate for the intervention message, that this message should be aimed towards friends, family, or current/future students, that students should make messages relevant to the lives of themselves or others, and that students should be specific about behaviors that exemplify the message. These requirements are deliberately chosen, designed to more deeply engage students with the intervention’s message. Because engagement is commonly understood through its cognitive, behavioral, and affective components (e.g., Fredricks et al., 2004), we use this same framework to organize why several of these requirements are considered integral to the success of saying-is-believing exercises.

#### Cognitive Component: Encouraging Recipients to Think Deeply about the Message

Interventions with writing prompts ask participants to not only reiterate, but also advocate for the message learned from the intervention to another person, often either a friend, family member, or current/future student. One reason this is done is to encourage people to actively, rather than passively, apply their knowledge, as active participation in constructing the message forces students to consider more deeply what the message itself is (Harackiewicz & Hulleman, 2010; Hiebert, 1992; Piaget, 1970). Second, it allows students to couch this information within memories related to the self, which are easier to remember thereafter compared to those that are more abstractly discussed (Bower & Gilligan, 1979; Putnam, 2015). For example, in utility-value interventions, students are asked to self-generate reasons the course material can be useful in the daily lives of themselves, friends, or family (Canning et al., 2018; Harackiewicz et al., 2016; Hulleman et al., 2010).

#### Behavioral Component: Encouraging Recipients to Connect the Message to Specific, Adaptive Practices

The theory of change behind wise interventions is that psychological beliefs will promote a variety of small, adaptive behaviors that can snowball into large academic gains through recursive processes (Walton, 2014). To help students translate the psychological belief into uptake of these behaviors, saying-is-believing exercises may also increase engagement with the intervention’s message by asking students to outline specific behaviors it should impact; evidence that the recipient understands what the message looks like in practice. For example, mindset interventions ask students to take in the growth mindset information learned from the scientific article and/or upperclassmen to their lives and write their own application examples for future students (Yeager et al., 2013, 2014). This activity can help translate a goal intention (e.g., “I will try harder in engineering because everyone goes through setbacks”) to an implementation intention (e.g., “Despite my low grade in engineering this semester, I will try harder by reviewing lecture materials, completing practice problems, and attending office hours because I believe that my knowledge can grow with effort;” Gollwitzer, 1993; Wormington et al., 2019). The actual implementation of the goal can become clearer when people generate proximal subgoals through writing because they can lay out a specific plan of how, when, and where it will be attained (Bandura & Schunk, 1981; Gollwitzer, 1999).

#### Affective Component: Framing the Cognitive and Behavioral Processes as Positive and Destigmatizing

Finally, in addition to the cognitive benefits of advocating the intervention’s message to others, framing the exercise as one that can help others may increase students’ affective engagement as well. Mindset, belonging, and difference education interventions often require that students present the message to current/future students who could learn from them (Murphy et al., 2020; Stephens et al., 2014; Walton et al., 2015; Yeager et al., 2016). As this requirement is forcing students to cognitively engage with the intervention message, it is also framing students as givers rather than receivers of the message. When students are in the position of helping others rather than needing help, they are more likely to feel positive emotions (Dunn et al., 2008; Harbaugh, 1998), which can thereby promote more academic engagement and performance (Carmona-Halty et al., 2021). Additionally, the thought of helping others through this exercise can reduce feelings of stigma because students are less likely to think of themselves as recipients of a persuasive appeal (Alvarez & van Leeuwen, 2015; Walton et al., 2015). Because affective engagement is positively related to cognitive and behavioral engagement (Archambault & Dupéré, 2017; Fredricks et al., 2004; Wang & Eccles, 2012), structuring the exercise so that it optimizes positive affect while reducing negative affect may actually be a crucial antecedent for students’ cognitive and behavioral engagement.

### Differences in Engagement During Saying-is-Believing Exercises

Although the current literature considers the saying-is-believing effect vital to the success of wise interventions, there is little research that examines to what extent they are engaging students with the intervention message, and whether some students may be more engaged than others. Saying-is-believing exercises have been reported to be a powerful technique in the wise intervention literature due to the theory of cognitive dissonance. Research on cognitive dissonance shows that individuals are motivated to align their attitudes, beliefs, and behaviors due to feelings of discomfort when those aspects are not psychologically consistent with one another (Festinger & Carlsmith, 1959; Festinger, 1957, 1962). That is, synchronic feelings are important for an individual’s life (Russo-Netzer & Icekson, 2020). However, implicit in the saying-is-believing effect line of reasoning is the assumption that participants in the intervention have similar experiences of dissonance. Namely, all participants in the intervention experience the intended cognitive dissonance. Scholars who study wise interventions believe that creating situations where individuals are asked to endorse the message of the intervention can build lasting changes in attitudes (Walton & Wilson, 2018). However, that cognitive dissonance is less likely to occur if the saying-is-believing exercise is not effectively engaging students cognitively, behaviorally, or affectively. As the origin of the term saying-is-believing attests, getting students more involved in the process can more strongly induce dissonance (George & Edward, 2009), and thereby lead them to experience different levels of cognitive dissonance (Soutar & Sweeney, 2003). The more deeply students become engaged in the task, the more likely they will be to experience dissonance and the psychological shifts that it typically induces. For example, if students in an engineering course are asked to write a letter to a prospective engineering student how understanding concepts related to stability, strength, and rigidity relates to their everyday lives such as the importance of how the building they are in is built, then students who make greater connections are more likely to experience the intended dissonance than those who make less connections.

In summary, we use an engagement framework for understanding prior literature on the requirements for how saying-is-believing exercises should be approached (e.g., Higgins & Rholes, 1978; Walton & Cohen, 2011). We hypothesize that the effectiveness of saying-is-believing exercises should depend on the extent to which cognitive, behavioral, and affective experiences take place. The framework of academic engagement has often been explored using three subcategories: affective, cognitive, and behavioral (Appleton et al., 2008; Fredricks et al., 2004). Cognitive experiences refer to the extent to which advocating for a particular position, such as finding engineering interesting or discussing tips on how to study well, changed someone’s attitudes and goals. Behavioral experiences capture the extent to which providing insights and tips to help students’ persistence in engineering and going through the process of engaging in the saying-is-believing exercise can change one’s own behavior. Lastly, affective experiences, the extent to which the participants have positive experiences engaging in the assigned activity (i.e., creating videos), can determine how much one endorses and applies the information to one’s own life at the end of their experience. The more students experience these different types of experiences, the more likely their attitudes, beliefs, and behaviors are to align because of the greater discomfort students felt engaging in the saying-is-believing exercise according to the theory of cognitive dissonance.

### Current Study

Given the importance of the saying-is-believing effect to intervention effectiveness, we designed this study to bridge the gap in the literature by understanding for whom, under what conditions, and how students describe the saying-is-believing effect. In the present study, we qualitatively investigated the ways in which students describe their experience creating YouTube videos (i.e., a saying-is-believing exercise) related to the saying-is-believing effect to address the following research questions (RQ):

- **RQ1:** What components do students believe engage them in a saying-is-believing exercise?
- **RQ2:** Are some students more engaged in a saying-is-believing exercise?

We anticipated the answers to these questions to vary depending on students’ affective, cognitive, and behavioral experiences creating YouTube videos about their engineering experiences.

## Method

### Participants

Participants were 14 undergraduates (12 males and 2 females) in a scholarship program for low-income transfer students from community college who want to pursue their baccalaureate in engineering (see Table 1). These participants were purposely selected because all students who received the scholarship experienced filming YouTube videos as part of their scholarship requirement. They were asked to film a total of four YouTube videos about their engineering experience, which were going to be shown to community college students.^1^ Participants were informed that their videos would be used to launch an intervention to promote community college students’ interest, course-taking, and persistence in the engineering major. The four different video topics were freely chosen by participants as long as the purpose of intervention was not explicitly stated to the audience because their videos were going to later be used for an intervention (e.g., I hope this video motivates you to keep on pursuing engineering; see Table 2 for video topics). Thus, the topics of the videos reflected what participants believed to be important for community college students to know. Some participants had the same two video titles as another participant because they were given the option to work with someone for two out of the four videos. Each participant was invited to schedule a one-on-one interview to discuss their experience filming YouTube videos after completion of filming four videos.

**Table 1:** *Participant information*

Participant Pseudonym Name	Age	Gender	Race/Ethnicity	Year at Current University
Ali	20	Male	White	Junior
Francis	21	Male	White	Junior
Parker	21	Male	Multi-Racial	Junior
Eduardo	23	Male	Hispanic/Latino	Junior
Adam	22	Male	Multi-Racial	Senior
Hai	20	Male	Asian	Junior
Michael	20	Male	White	Junior
Kristin	20	Female	Asian	Junior
Rani	21	Male	Asian	Junior
Guanyu	22	Male	Asian	Junior
Alec	22	Male	Hispanic/Latino	Junior
Lukas	24	Male	Hispanic/Latino	Junior
Hillary	23	Female	White	Junior
Otto	22	Male	Multi-Racial	Junior

*Note.* All students transferred from community college to a four-year university. Year at current university refers to their year standing at the four-year university

**Table 2:** *Video title filmed by participants*

	Video Title			
Participant Pseudonym Name	Video #1	Video #2	Video #3	Video #4
Ali	Introducing Ourselves and Answering Some Engineering Questions	How to Succeed in Classes: Study Tips and Environment	Study Tips During the Pandemic	Financial and Scholarships and their Benefits
Francis	How I Got Into Mechanical Engineering and Why I Chose It	Gaining Experience & Professional Development in the Realm of Engineering	My Journey From Community College to University	How to Approach Engineering Projects
Parker	Transferring From Community College To University	How To Get Research Positions In The [University] System	How To Get A Summer Internship	Study Methods And Tips For University
Eduardo	Q&A! Answering FAQs Transferring Mechanical and Biomedical Engineering Students Have	Studying Advice for Transitioning from Community College to University	How Did I Get Here?	My Take on COVID-19
Adam	How to be a Successful Engineering College Student pt. 1: Time Management	How to be a Successful Engineering College Student pt. 2: Study Tips	All About Teamwork	Work From Home: My Experience and Advice
Hai	How Kindness Has Led Me To Engineering…	How To Get Help & Who To Get Help From	How To Stay Involved & Take Advantage Of On-Campus Opportunities	How Expectations Stack Up To Reality…
Michael	Introducing Ourselves and Answering Some Engineering Questions	How to Succeed in Classes: Study Tips and Environment	Social Life and Making Friends at [UNIVERSITY NAME]	Engineering Student Experience during the COVID-19 Pandemic
Kristin	Choosing a Major and How to Prepare for a Transfer	First Quarter at [UNIVERSITY NAME]	Overcoming Adversity with Preparation and Motivation	Applying to Internships and How to Write Cover Letters and Resumes
Rani	My Community College Experience	Transitioning Into University	How To Be A Successful Student	Answering Common Questions About Engineering Majors
Guanyu	Study Tips and Habits	Advice for Research and Internships	Resources at [UNIVERSITY NAME]	Things I Wish I Knew Before Coming to University
Alec	How to be a Successful Engineering College Student pt. 1: Time Management	How to be a Successful Engineering College Student pt. 2: Study Tips	Connecting In Your Major	Finding Your Career Path
Lukas	First Generation Engineering Chronicles: Introduction	First Generation Engineering Chronicles: Networking	First Generation Engineering Chronicles: Studying at University	First Generation Engineering Chronicles: Research and Graduate School
Hillary	Q&A! Answering FAQs Transferring Mechanical and Biomedical Engineering Students Have	How I Studied This Quarter at [UNIVERSITY NAME] as a Biomedical Engineering Major: Classes, Advice, and Tips	Choosing My Major and School	My COVID-19 Experience
Otto	All About the Mechanical Engineering Major and Why I Chose It	Preparing for Engineering Classes at [UNIVERSITY NAME]	Staying Involved as an Engineering Major	Answering Common Engineering Questions

*Note*. Each participant filmed a total of four YouTube videos

We analyzed semi-structured interviews from 14 students out of the 17 students who completed filming all four YouTube videos. Of these, four (29%) identified as Asian; four (29%) identified as White; three (21%) identified as Hispanic/Latino; and three (21%) identified as multi-racial. Three of students were not able to be followed-up for an interview because they were difficult to stay in contact with after the end of the academic term or after graduation. The age ranged from 20–24 (*M* = 21.5; *SD* = 1.29). Most participants identified as junior standing at their current four-year university (13 juniors and 1 senior).

### Interview Procedures

Each participant provided consent prior to data collection. Participants were interviewed online using Zoom during the spring and summer of 2020. This study focused on the transfer community college students in the scholarship program to gain insight into their unique perspective on filming YouTube videos about their engineering experiences related to the saying-is-believing effect. The interviews were semi-structured to allow for probing further details of a question and following a more open-ended conversation style. The interview script ensured the coverage of topics related to the choice of video topics, emotions, attitudes, goals, thoughts, and behavioral change they experienced as they created videos about their engineering experience. Interviews began with asking a broad question about their experience filming their videos: “What was your experience like being a YouTuber?” They were then probed in-depth regarding the emotional (e.g., “Were there any aspects of this project that you enjoyed/disliked?”), cognitive (e.g., Did you have any goal for the project), and behavioral (e.g., Did this process of creating YouTube videos affect your experiences in engineering courses?”) experiences, in order to understand how the different facets of their experiences can be related to the saying-is-believing effect.

Interviews of approximately one hour were conducted by the first and second authors of this paper. Interviewers met with each other to revise the interview script and practice interview skills to enhance reliability and fidelity of the procedure. Also, they met weekly to debrief each other and address any concerns with the interviews. Research assistants transcribed the interviews from the audio recordings.

### Coding and Analyses

Both inductive and deductive approaches were used to identify patterns within the data using Microsoft Word and Excel. An inductive approach refers to creating codes that arise from the data, whereas a deductive approach refers to using predetermined codes from theory (Saldaña, 2013). In the first stage of coding, the first author read through all 14 transcripts in entirety to begin to understand patterns in the data. As discussed in the introduction, three different types of experiences were used to determine the extent to which individuals differ on the saying-is-believing effect (i.e., affective, cognitive, and behavioral experiences; see Table 3). Affective experiences are defined as a person’s emotional experiences during the process of creating YouTube videos about their engineering experience. Within affective experiences, there are positive (e.g., excited about helping others) and negative (e.g., frustrated with lighting and camera angle) affective experiences. Cognitive experiences are defined as a person’s attitudes and goals with regard to creating their YouTube videos. Within cognitive experiences, there are changes in perception about self-identity (e.g., feelings of being an engineer) and self-engagement in the project (e.g., feeling like one can accomplish more than now through creating videos). Lastly, behavioral experiences are defined as behaviors that might change as a result of being involved in the video creation process itself. Within behavioral experiences, there are soft skills learned (e.g., editing, filming, etc.), academic-related skill indirectly learned from video content (e.g., teamwork and time management), and academic-related skills directly learned from video content (e.g., learning about and now using an app after talking about that app in a video).

**Table 3:** *Codebook for each type of experience*

Experience	Definition	Example
*Affective Experiences*	Affective experiences are defined as a person’s emotional experiences during the process of creating YouTube videos about their engineering experience	
Positive Affective Experiences	A positive affective experience refers to a positive emotional experience, in regard to creating videos (e.g., feeling good that they are helping others and have the ability to share experiences with others	Lukas: “I really enjoyed the idea of being able to film our thoughts, and be able to share them with whoever.”
Negative Affective Experiences	A negative affective experience refers to a negative emotional experience, in regard to creating videos (e.g., feeling like there was no point in creating videos because they did not believe in the value or environmental factors like lighting that got in the way of filming)	Michael: “No matter how hard I tried, I still felt like they’re kinda like they won’t make a difference to anybody.”
*Cognitive Experiences*	Cognitive experiences are defined as a person’s attitudes and goals with regard to creating their YouTube videos	
Changes in Perception about Self-Identity	A change in perception about self-identity refers to how an individual views oneself differently after creating videos (e.g., learning to not compare oneself to others, cemented the feelings of being an engineer, and re-evaluate interests and priorities)	Alec: “The videos helped me to like to stop and take a break and see what I am doing. And is this what I want to keep on doing?”
Changes in Perception about Self-Engagement in the Project	A change in perception about self-engagement in the project refers to how an individual might engage differently in the project after creating videos (e.g., inspired to give back as others had helped them or reflecting on their video performance compared to prior videos filmed)	Adam: “I just kinda felt that like the last video, I was just kinda dry on like what to talk about. I still think I shared good information, but I don't feel as like, ‘Wow, I was so insightful’ as I feel about like maybe my first, second, and third video.”
*Behavioral Experiences*	Behavioral experiences are defined as behaviors that might change as a result of being involved in the video creation process itself	
Soft Skills Learned	Soft skills refer to technical skills, such as filming and editing videos	Kristen: “While editing, I can see things that I need to work on with public speaking because that’s a very important skill to have when you’re working in a professional environment.”
Academic-Related Skills Indirectly Learned from Video Content	Academic-related skills indirectly learned from video content refers to learned skills through the process of creating videos rather than video content presented in the videos (e.g., teamwork and time management)	Ali: “Working with another student through this, you know, it was a new experience for both of us. So, we kinda had to work together. You know, setting up the dates and whatnot. Like talking to each other about what we want to say. That's going to help in the future with like teamwork. As an engineer, you're most likely going to be working with a team. You know what I mean. You're never alone.”
Academic-Related Skills Directly Learned from Video Content	Academic-related skills directly learned from video content refers to learned skills through talking about a particular topic in one’s video rather than just involvement in creating videos (e.g., learning about and now using an app after talking about that app in a video)	Hillary: “This quarter, in one of my classes, the professor told us that there were some senior students with their own senior project, but they needed help to make an app for it. Our professor just mentioned it in class, and I followed up to help them. I tried to be more active or try to participate in more things.”

From the three deductive codes of experiences (i.e., affective, cognitive, and behavioral), the first two authors and research assistants identified codes using in-vivo (i.e., literal or verbatim words or phrases used by participants) and descriptive (i.e., summarizing words or phrases used by participants) coding (Saldaña, 2013). Each in-vivo and descriptive code was copied into a Microsoft Word document organized by three categories of experiences. The research team chose codes that were mentioned most frequently. Then in the second stage of coding, codes were refined based on the interview data and discussions amongst our research team. Coders independently coded each transcript before reconciling discrepancies. Furthermore, during all coding phases, coders wrote detailed analytic memos about patterns of experiences filming videos related to the saying-is-believing effect and changes made to collapse or divide codes.

To investigate how much a participant differs on the saying-is-believing effect compared to others after creating videos, we used both inductive and deductive approaches. We first used the conceptual framework of the saying-is-believing effect to determine what the extreme ends of each level might look like. For example, someone who exhibits a high level of the saying-is-believing effect might have a positive change in multiple behaviors (e.g., attending more networking events, getting more involved in research, etc.) from conveying the importance of connecting with people in the field of engineering as future engineers in their videos. On the other hand, individuals who exhibit a low level of the saying-is-believing effect might neither experience a change in attitude nor more strongly endorse a message that one has been advocating in their videos. This conceptual framework of the two opposite ends of the saying-is-believing effect laid the groundwork for placing students into different categories of the saying-is-believing effect.

Then an inductive approach was used to categorize each participant into low level, medium–low level, medium–high level, and high level of saying-is-believing effect relative to others based on their affective, cognitive, and behavioral experiences using the data in its entirety as well as analytic memos. Coders determined how affective, cognitive, and behavioral experiences differed for each level of the saying-is-believing effect (i.e., low level, medium–low level, medium–high level, and high level). Each main sub-category of affective, cognitive, and behavioral experiences was included: positive and negative affective experiences, change in attitudes about self-identity, change in attitudes about self-engagement in the project, soft skills learned, academic-related skills indirectly learned from video content, and academic-related skills directly learned from video content.^2^ Coders independently categorized participants using a Microsoft Excel Sheet and discussed their codes to reconcile discrepancies.

## Results

### What Components Did Students Believe Engaged Them in A Saying-is-Believing Exercise?

Coding for evidence of students’ affective, cognitive, and behavioral engagement in the exercise, several representations of engagement emerged; these are listed and defined alongside example quotes in Table 3. For affective experiences, the main subcategories found were positive and negative reasons from creating videos. Whereas positive affect is hypothesized to be an indicator of engagement, negative affect was hypothesized to be an element of disengagement. For cognitive experiences, the main subcategories found were change in perceptions about self-identity and self-engagement in the project. For behavioral experiences, the main subcategories found were soft skills learned, academic-related skills indirectly learned from video content, and academic-related skills directly learned from video content.

### Were Some Students More Engaged in A Saying-is-Believing Exercise?

**Fig. 1:**
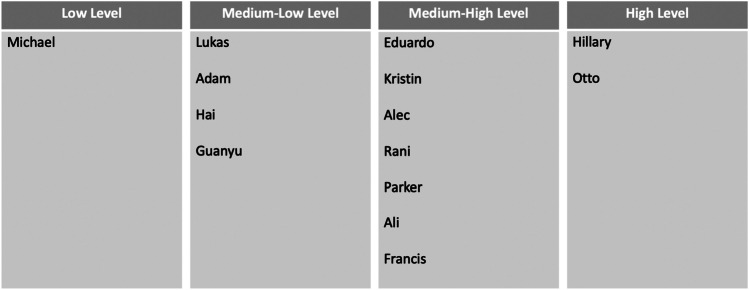
*Levels of the saying-is-believing effect*

The components of engagement outlined above did not appear equally across all students as they discussed their experience with the saying-is-believing exercise. Taking an additive perspective to understanding engagement, we considered that students were more engaged if they reported experiencing more of the components of engagement outlined above. This created four categories of engagement: low, medium–low, medium–high, and high level. Table 4 outlines the configurations of engagement that define each category, along with how many students in the sample fell into each category. We describe cases of participants from each level of the saying-is-believing effect utilizing categories from affective, cognitive, and behavioral experiences. Results showed that participants varied on where they fell into the different categories (refer to Fig. 1). Participants endorsed a low level, medium–low, medium–high, to high level of the saying-is-believing effect. The majority of participants fell into the medium level saying-is-believing effect, which we distinguish between medium–low and medium–high level of the saying-is-believing effect.

**Table 4:** *Different levels of the saying-is-believing effect*

	Levels of the Saying-is-Believing Effect				
	Low Level	Medium–Low Level	Medium–High Level		High Level
Affective Experiences					
Positive Affective Experiences		✓	✓	✓	✓
Negative Affective Experiences	✓	✓	✓	✓	✓
Cognitive Experiences					
Changes in Perception about Self-Identity		✓	✓	✓	✓
Changes in Perception about Self-Engagement in the Project		✓	✓	✓	✓
Behavioral Experiences					
Soft Skills Learned			✓	✓	✓
Academic-Related Skills Indirectly Learned from Video Content				✓	✓
Academic-Related Skills Directly Learned from Video Content					✓
Number of Students in Each Level	1	4	7		2

*Note*. Checkmark represents the presence of that theme or experience. Negative affective experience is strongest in the low level compared to the other levels

#### Low Level of the Saying-is-Believing Effect

Student(s) who were categorized as showing low levels of the saying-is-believing effect experienced only negative affective experiences (refer to Table 4). They reported no experiences that fit into either cognitive or behavioral experiences. In contrast, although student(s) who showed higher levels of the saying-is-believing effect also reported negative affective experiences, the nature of these negative experiences differed. Student(s) with a low level of the saying-is-believing effect felt negative affective experiences from engaging in the activity itself. They did not see the value or larger goal of creating YouTube videos nor did they believe that sharing one’s engineering experience will help others. For example, Michael, a student who has a low level of saying-is-believing effect said, “No matter how hard I tried, I still felt like they’re kinda like they won’t make a difference to anybody.” Other students with higher levels of the saying-is-believing effect expressed that they “saw this experience as an opportunity to help others” or “felt part of something greater than just a video,” but Michael felt that the videos he made were pointless because he did not believe that watching them will change anything. A key factor for someone in the low level is only feeling negative affective experience, leading them to not have a change in perceptions about self-identity and self-engagement in the project. Yet, both groups were similar in the sense that they felt negative affective experiences from the process of filming such as feeling like it can be time consuming to film videos while balancing schoolwork, presenting oneself to others via video, and filming with environmental constraints.^3^

#### Medium–Low Level of the Saying-is-Believing Effect

Student(s) who were classified under the medium–low level of the saying-is-believing effect experienced positive and negative affective experiences as well as a cognitive change in perceptions about self-identity and self-engagement in the project (refer to Table 4). Compared to students in the low level, students in the medium–low level of the saying-is-believing effect felt positive affective experiences. For instance, students felt positive affective experiences from helping others through sharing their experience, learning particularly with repetition, freedom to express oneself, and bonding with peers. Adam, a student who has a medium–low level of the saying-is-believing effect expressed, “l liked the idea of like sharing—like stuff that happened to me, so people don’t make the same mistakes” It is worth noting that student(s) with a medium–low level of the saying-is-believing effect felt affective experiences that could simultaneously represent balances of both positive and negative experiences, such as feeling challenged to come up with good topics. For example, Adam also described the challenges he had with finalizing video topics: “One of the harder aspects is like thinking about what to say, like what are the most valuable things to share, like what are good topics. I think that was like the hardest part.” The combination of recognizing their choice of video topics was important, yet challenging, suggested that even as positive affect can encourage students to attempt the task, feeling frustrated by the task’s difficulty can produce negative affective experiences.

Student(s) in the medium–low level also had positive cognitive experiences. They had changes in perceptions about self-identity. First, these student(s) reflected on themselves throughout the process of creating videos, such as learning to not compare oneself to others after creating videos. Student(s) realized how much they accomplished by talking about their engineering experience. For example, Lukas who had trouble comparing himself to other people described how the videos helped him see that he is doing just fine:

[I realized] that not everyone has the same workflow as you, and like not everyone has the same requirements and additional add-ons that we may have. So, we really shouldn’t be comparing ourselves to other people and stuff will build up—anxiety and stuff. It helps me just try to like not care about comparing myself to everyone’s workflow.

Lukas, a student with medium–low level of the saying-is-believing effect was able to reflect on his strengths through the video creation process, which in turn, aided him to understand that he should not compare himself to others.

Second, these student(s) had a change in perception about self-engagement over the project. They felt inspired to give back as others had helped them and reflected on their video performance compared to each other. Lukas talked about his appreciation for all those who had helped guide him to where he is today. He said, “I want to highlight each one of these individuals and how they led me to opportunities that have made me more competitive.” They also used their previous video performance to gauge their current video performance. The following excerpt highlights Adam’s experience of comparing his own videos to each other:

I just kinda felt that like the last video, I was just kinda dry on like what to talk about. I still think I shared good information, but I don't feel as like, ‘Wow, I was so insightful’ as I feel about like maybe my first, second, and third video.

Although Adam was able to reflect on his weakness, there was no thought of working immediately to alleviate the problem. The desire to work on one’s weakness is one that differentiates between medium–low and medium–high saying-is-believing effect. Overall, students who had positive cognitive experiences without changes in behavioral experiences lead them to be medium–low, but positive cognitive experiences with changes in behavioral experiences lead them to be medium–high. Further, compared to students with a low level of the saying-is-believing effect, students with a medium–low level had the presence of other affective experiences than just negative affective experiences as well as cognitive experiences.

#### Medium–High Level of the Saying-is-Believing Effect

Student(s) who were categorized under the medium–high level of the saying-is-believing effect experienced positive and negative affective experiences, a cognitive change in perceptions about self-identity and self-engagement in the project, and behavioral experiences of soft skills learned, and academic-related skills indirectly learned from video content (refer to Table 4). Compared to students with a medium–low level, students with a medium–high level the added component of behavioral experiences: soft skills learned or academic-related skills indirectly learned from video content. Students with a medium–high level of the saying-is-believing effect felt that certain aspects of their attitude related to their self-identity had changed as well as their behaviors. For example, those with a medium–high level reported being able to reflect and start to enact a plan to overcome their weakness, evaluate their interests and priorities, reinforce their career goals, and cement the feeling of being an engineer. Compared to Adam who has a medium–low level, Francis who has a medium–high level of the saying-is-believing effect reflected on his weakness of public speaking through watching himself speak in his own videos and planned to overcome this weakness by learning how to make his videos better. Francis states learning how to communicate to an audience more clearly and concisely:

I think that something else I’ve learned is the way I speak. What I realized is when I'm on my first few videos, I think that I wasn't clear enough, and that’s something I've realized. And like my communication skills, I think that’s still an important part of engineering, overall.

Moreover, student(s) in the medium–high level found that the videos helped cement the feeling of being an engineer because “usually engineers don’t talk about their experiences too much and it’s usually about their projects,” Francis stated. Doing the YouTube videos allowed them to share “their school, or about their life, or experiences,” which reaffirmed that being an engineer is not just about what projects one is working on but involves multiple facets of their life. For others, creating the videos reinforced their career goal. Ali, a student who has medium–high level of the saying-is-believing effect, described how creating the videos reinforced his goals of going to medical school: “I feel like it more cemented the idea, you know what I mean, like I talked about the cost of school, for example.” The process of creating YouTube videos helped Ali reinforce his reasons for wanting to attend medical school after graduation and reminded of him to work harder to reach his goals. Similarly, other students found that creating videos helped evaluate their interests and priorities. Alec who has a medium–high level mentioned that “The videos helped me to like to stop and take a break and see what I am doing. And is this what I want to keep on doing?”.

The medium–high level students also reported learning soft skills and academic-related skills indirectly from video content. The soft skills mentioned included editing, communication, and self-presentation. The academic-related skills indirectly learned from video content included learning about time management and teamwork. For example, Kristen said,

While editing, I can see things that I need to work on with public speaking because that’s a very important skill to have when you’re working in a professional environment.

Kristen learned soft skills like editing, which helped her realized that she could improve her public speaking skills. She was also able to manage her time better because of the time needed to film videos: “I had to teach myself how to better time manage.”

Another example of a student from a medium–high level expressing academic-related skills indirectly learned from video content comes from Ali. He discussed how this experience of creating videos helped him gain teamwork skills:

Working with another student through this, you know, it was a new experience for both of us. So, we kinda had to work together. You know, setting up the dates and whatnot. Like talking to each other about what we want to say. That's going to help in the future with like teamwork. As an engineer, you're most likely going to be working with a team. You know what I mean. You're never alone.

#### High Level of the Saying-is-Believing Effect

Student(s) who were categorized under the high level of the saying-is-believing effect experienced positive and negative affective experiences, a cognitive change in perceptions about self-identity and self-engagement in the project, and behavioral experiences of soft skills learned, academic-related skills indirectly learned from video content, and academic-related skills directly learned from video content (refer to Table 4). The main difference in the high level saying-is-believing from the other levels is the presence of academic-related skills directly learned from video content. Academic-related skills directly learned from video content referred to learned skills through talking about a specific topic in one’s video rather than just being involved in creating videos. Students in this category displayed the highest level of saying-is-believing because discussing topics to inform and teach current and future engineering students changed their own behavior. For example,

Hillary: This quarter, in one of my classes, the professor told us that there were some senior students with their own senior project, but they needed help to make an app for it. Our professor just mentioned it in class, and I followed up to help them. I tried to be more active or try to participate in more things.

Otto: I definitely mentioned career fairs, and back in community college, we had some career fairs, but I didn’t really go because I thought of them as not really useful. But after making this video, I thought of it as a good way to build connections and to meet new people and being part of an experience—having conversations with professionals and getting a feel of that formal talk.

Both Hillary and Otto experienced behavioral changes as a result of talking about the importance of research involvement and career fairs. They were able to reflect on their weaknesses through talking about tips for success to current and future students and enacted upon them.

## Discussion

Saying-is-believing exercises are considered fundamental to the success of wise interventions for their role in helping students engage with and internalize the intervention message (Aronson et al, 2002; Higgins & Rholes, 1978). But engagement itself is a nebulous construct, and it can vary from student to student. This qualitative investigation therefore sought to understand the components that can comprise engagement in a saying-is-believing exercise and investigate how differently students may experience that engagement. Below, we discuss our findings’ theoretical implications, suggestions for building better interventions, and what will be needed for researchers to incorporate saying-is-believing engagement measures into their analyses of intervention effectiveness.

### Implications for the Theory Behind Saying-is-Believing Exercises

Delineating the components of engagement in a saying-is-believing exercise first confirms the study’s premise that some students may be more engaged than others. For example, results showed that students varied on the level of engagement in the saying-is-believing exercise of creating YouTube videos: low, medium–low, medium–high, and high level. We found that students who have higher levels of engagement endorsed all affective, cognitive, and behavioral experiences compared to those with lower levels of engagement. This difference in intervention effect connects well with Vygotsky’s notion of the Zone of Proximal Development (ZPD; 1978). The ZPD refers to a zone of learning increases that an individual can accomplish with the help of someone else. On the other hand, the Zone of Achieved Development (ZAD) refers to a zone that an individual can accomplish on their own or without the help of someone else. In intervention work, Vygotsky (1978) argued that an individual should be within the ZPD to effectively change. In line with this reasoning, students with low levels of the saying-is-believing effect might not be in the ZPD to gain benefits from engaging in the saying-is-believing activity of creating videos. Or perhaps, students with low self-efficacy are less likely to feel that they can benefit (Eccles-Parsons et al., 1983; Eccles & Wigfield, 2020). Whereas students who are in the ZPD do not have any benefits to earn from doing the saying-is-believing activity because they already can do this on their own (i.e., a ceiling effect). These particular individuals are probably endorsing the believing-is-saying effect rather than the saying-is-believing effect because they already believe in the advocated messages. Initial measures of where individuals start seem to be important factor for how much one gain advantages from doing the saying-is-believing activity. Thus, future studies should measure initial starting points before conducting an intervention. This assessment can help us focus on individuals in the ZPD for the intervention.

Second, we see evidence that validates the theory of change behind psychological interventions. Despite the many potential combinations of affective, cognitive, and behavioral engagement that could have occurred, it is meaningful that we only saw a few combinations that suggested there may be patterns in how different forms of engagement co-occur. For instance, behavioral engagement was always observed by those also experiencing both affective and cognitive engagement. Psychological interventions are built on the idea that psychological beliefs are crucial for spurring changes in behaviors (Aronson et al., 2002; Stephens, et al., 2014; Walton & Cohen, 2007, 2011; Walton & Wilson, 2018). It would therefore have been strange for those going through a saying-is-believing exercise to have discussed behavioral considerations without corresponding cognitive changes. Preliminary analyses, however, show that this did not always mean participants linearly discussed cognitive changes before behavioral changes. Some of them discussed behavioral engagement first, before reflecting on changes in perception about self-identity and self-engagement in the project (i.e., cognitive). However, this should not necessarily be surprising considering it reflects the way that cognitive dissonance operates to create psychological change. Future studies should examine the relations between affective, cognitive, and behavioral experiences. We discuss this point further in the future directions section of the paper.

### Implications for Building Better Interventions

Psychological interventions are assumed to impact academic outcomes by impacting students’ behavior. It is therefore crucial to notice that in our study, behavioral engagement, was always accompanied by positive affective engagement and cognitive engagement. Both affective and cognitive engagement during participants’ experience creating videos seemed to be needed for “fertile growth” (i.e., translation of the psychological message into behavior; Walton & Yeager, 2020). We recommend structuring saying-is-believing exercises to explicitly elicit affective, cognitive, and behavioral engagement to scaffold students’ internalization of the message (e.g., offer examples like “Even though I learned that everyone struggles, I still have times when I do not feel like I belong; in these situations, I will do X, Y, and Z”). To foster cognitive engagement, specifically, we encourage intervention designers to create saying-is-believing exercises that can help change perceptions related to their self-identity, in particular those that provide the opportunity to evaluate interests and priorities, reinforce career goals, and cement the feeling of being an engineer. Future research may consider various prompts from among these suggestions to evaluate their effects in more detail.

Additionally, supporting positive affective engagement may be especially important to attend to. One student categorized in the low level bemoaned that they did not think their videos could really help others, and thereafter did not seem to experience any of the intended changes in their thinking or behaviors that the intervention was designed to stimulate. Following Strack and Deutsch (2004), associative evaluations can explain how perceptions change. If students have a positive affective reaction to the saying-is-believing activity of creating videos, then they might be more likely to benefit from engaging in the saying-is-believing activity because of a likely suggestion that transforms a thought to “I like creating videos.” Whereas if students do not have a positive reaction to the saying-is-believing activity, they are not as likely to benefit from the saying-is-believing activity because there is no initial push to create a positive attitude change. Intervention researchers should ensure that the purpose of the exercise (e.g., helping current and future students adapt) is clear and attainable. Researchers may be more successful encouraging students to provide details about their thinking and behavior; for instance, if they explain in the instructions that providing such details serve the purpose of making their message more helpful and convincing for other students. As we only had one student in this sample who represented this phenomenon, more work may be needed to identify various sources of students’ skepticism about the benefits of deeply engaging in saying-is-believing exercises.

### Implications for Analyzing Treatment Heterogeneity

Aforementioned, intervention researchers are moving towards understanding heterogeneity in treatment effects (McPartlan et al., 2020; Walton & Yeager, 2020; Yeager et al., 2019; Yeager & Dweck, 2020). Our study found support for the notion that positive affective engagement with the saying-is-believing exercise may be an important first step toward engagement with the intervention. But although cognitive and behavioral engagement may be more quickly measured through text analysis (Harackiewicz et al., 2016; Hecht et al., 2019; Priniski et al., 2019), affective engagement may not be as readily discernable within the text itself. Future studies might therefore consider validating short instruments for measuring affective engagement after the exercise. Contextually relevant items may include students’ enjoyment of the exercise and their belief that their efforts are likely to help others.

### Limitations and Future Directions

While the results provided us useful insights on heterogeneous experiences with the intervention and the process of the different levels of the saying-is-believing effect, inherent limitations within the current study should also be acknowledged. We interviewed a total of 14 participants. Therefore, findings cannot claim that all possible patterns of affective, cognitive, and behavioral experiences that determine the difference between various levels of the saying-is-believing effect were found. For instance, our study found that negative affect appeared for everyone. This finding might not generalize to shorter interventions because we asked students to do a considerable amount. Whereas students in this study often lamented the involvement required to make multiple videos, shorter exercises may not generate negative feelings for most students. Still, concerns over the generalizability of the exact engagement configurations found would not necessarily take away from the main findings of this study. Specifically, that affective, cognitive, and behavioral experiences are useful for understanding students’ engagement with saying-is-believing exercises, and that some students experience more engagement than others. Future studies should attempt to understand how findings from this study generalize across different saying-is-believing exercises. The present study focused on the context in which students were asked to film a series of four YouTube videos for engineering students.

Additionally, our study did not consider the role of particular demographic characteristics that might affect where individuals are on the saying-is-believing effect continuum. Previous literature on wise interventions suggest that treatment-effects differ by demographic characteristics of the individual, such as race, gender, or first-generation status (Sisk et al., 2018). Perhaps, first-generation college students were more likely to gain benefits from the saying-is-believing activity of creating videos compared to continuing-generation college students because the initial feeling of wanting to help others was stronger in this group (Allen et al., 2015). Future studies should test this effect. Although our study did not examine whether participants’ demographic characteristics impacted the extent to which they endorsed the saying-is-believing effect, we believe that it laid the groundwork for understanding the differential impact and process of the saying-is-believing effect. We believe that future studies should follow-up with conducting more interviews with students from different backgrounds, in order to understand the differences and similarities across groups.

Moreover, although this study did not directly test whether different forms of engagement effectively build on each other when engaging in a saying-is-believing exercise, findings indicated that some students talked about cognitive engagement supporting behavioral engagement, and vice-versa. Some participants first experienced a change in their perceptions (i.e., cognitive experiences), which led them to a behavioral change. Others first experienced a behavioral change before experiencing a change in their perceptions (i.e., cognitive experiences). Finally, there were some participants that experienced an iterative cycle of behavioral and cognitive experiences. In alignment with psychological research, people often face multiple iterations of affective, cognitive, or behavioral experiences (Nevis, 1997). Thus, future research should aim to closely examine the process of the saying-is-believing effect using categories from affective, cognitive, and behavioral experiences. In particular, more work will be needed to understand whether affective, cognitive, and behavioral measures of engagement do indeed operate additively and should be aggregated to create an overall metric of engagement. Or, simply the presence of one component of engagement, such as behavioral, is sufficient for predicting different students’ outcomes. This work may help refine this study’s ordinal scale representing engagement, suggesting additional scale points be added or proposing something more akin to an interval scale.

Finally, the current study did not take into consideration how individuals might vary on the time lapsed to benefit from the saying-is-believing activity. Perhaps, some individuals on the medium–low or medium–high level are not in the high level of the saying-is-believing effect because they need more time to gain benefits from creating videos. Future studies should consider varying the time lapsed after completing the saying-is-believing activity, in order to subsequently test their cognitive and behavioral changes or outcomes.

### Conclusion

Despite the limitations, to our knowledge, this is the first study to qualitatively examine the process of the saying-is-believing effect within the education wise intervention literature. Findings from this study can inform scholars who are looking to enhance the effectiveness of their intervention through careful construction of a saying-is-believing activity. Furthermore, this study provides valuable insights as to why some individuals might have not gained positive outcomes from a wise intervention. In order to create effective interventions, creators should think about the heterogeneous effects of an individual before assuming that the saying-is-believing activity will induce positive changes. Although more work is needed, we believe these insights will open the door to more nuanced investigations of saying-is-believing exercises. We hope that leveraging an engagement framework will be helpful for building theoretical models for saying-is-believing exercises, understanding heterogeneity in intervention effectiveness, and implementing more effective interventions overall.

## Data Availability

Data from the current are available from the corresponding author upon reasonable request.

## Footnotes

1 This saying-is-believing exercise of creating more than four videos is more than required for a typical wise intervention. However, we believe that the extensiveness of this procedure can give much better insight into the endpoints of a saying-is-believing effect.

2 Sub-categories of affective, cognitive, and behavioral experiences were finalized during the second stage of coding.

3 Environmental constraints that might make filming difficult: trying to film during the day to get optimal lighting, remembering previous placement of the camera to get the right framing, or working around roommates’ schedule to reduce interruptions.
